# Two Cases of Primary Spinal Glioblastoma in Adults Treated With Multimodality Therapy

**DOI:** 10.7759/cureus.32272

**Published:** 2022-12-06

**Authors:** Ella Mae Cruz-Lim, François Germain, Delia Sauciuc, Benjamin Mou

**Affiliations:** 1 Department of Radiation Oncology, BC Cancer Agency Sindi Ahluwalia Hawkins Centre for the Southern Interior, Kelowna, CAN; 2 Department of Radiation Oncology, Zamboanga City Medical Center, Zamboanga City, PHL; 3 Department of Medical Oncology, BC Cancer Agency Sindi Ahluwalia Hawkins Centre for the Southern Interior, Kelowna, CAN

**Keywords:** spinal cord glioblastoma, primary spinal cord tumors, adults, surgery, chemotherapy, radiation therapy, spinal glioblastoma

## Abstract

Primary spinal glioblastoma (GBM) is a rare disease entity with no established standard treatment. We present two cases of primary spinal GBM initially presenting with motor-sensory deficits and back pain. Management varied in that the first patient received subtotal resection followed by radiation therapy, while the second patient underwent gross total resection followed by radiation therapy and temozolomide. The first patient died from hypoxemia secondary to disease progression affecting diaphragmatic motion three months after diagnosis. The second patient progressed intracranially and died 7.4 months after diagnosis. There is no standard of care for primary spinal GBM, so treatment should follow a multidisciplinary discussion focused on patient-specific goals. These cases highlight the poor prognosis of primary spinal GBM despite different treatment approaches, necessitating accurate reporting of all similar cases to help improve knowledge and management of this rare malignancy.

## Introduction

Primary spinal glioblastoma (GBM) is a very rare malignancy with a poor prognosis and scant evidence of appropriate treatment. Unlike intracranial glioblastoma, primary spinal GBM accounts for only 0.2% of glioblastoma cases and 1.5% of primary spinal cord tumors, with only about 200 cases reported in the literature, making opportunities to improve management extremely challenging [1-2]. We herein present two cases of primary spinal GBM, including clinical presentation, radiologic findings, and treatment outcomes. 

## Case presentation

Case one

**Figure 1:**
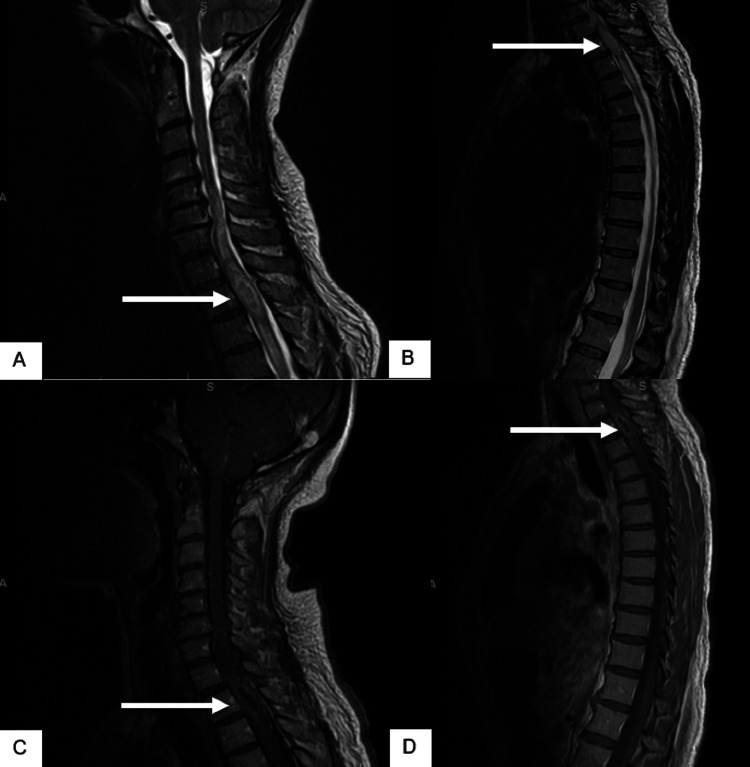
MRI of the cervicothoracic spine, sagittal view (A-B) T2-weighted and (C-D) T1-weighted post-contrast images showing an intradural lesion (white arrows) extending from the C5-T5 level.

A 54-year-old female retired government worker initially presented with numbness of her thoracic dermatomes associated with thoracic back pain localized around the T1-T5 spinal region. She presented to the emergency department, where an X-ray showed unremarkable results. The following day she noted paresthesia and weakness of the lower extremities causing difficulty with ambulation, as well as urinary retention. She was admitted to the hospital, where corticosteroids were immediately administered. Magnetic resonance imaging (MRI) demonstrated an intradural T2 cord abnormality extending from the bottom of C5 to the bottom of T5 with some post-contrast rim enhancement (Figure 1). The MRI findings were concerning for either metastatic disease or primary spinal cord malignancy. An MRI of the head revealed the absence of any intracranial mass.

Four days after the onset of paraplegia and dexamethasone intake, the patient still did not experience any improvement in her symptoms. The patient was Zubrod performance status 3 with 0/5 strength in bilateral lower extremities. She lacked any sensation from the T4 level downwards. She described some occasional discomfort in her back but denied any pain. The rest of her systemic physical examination was normal.

Upon multidisciplinary team discussion, it was decided that emergent radiation therapy would not benefit her situation. The team decided to obtain a histological diagnosis to better guide therapeutic management.

Six days after the onset of her symptoms, the patient underwent T1, T2, and partial T3 laminectomy and microsurgical resection of the spinal cord tumor. Postoperatively, American Spinal Injury Association (ASIA) exam showed a score of T3 ASIA A with normal upper extremity motor strength and 0 lower extremity motor strength. There was still no sensation from the T4 level downwards, with no zone of partial preservation.

The pathology from this procedure demonstrated glioblastoma, World Health Organization Grade IV, IDH-wildtype with no H3K27 mutation. The medical oncology service did not recommend concurrent temozolomide with radiation therapy due to the limited data to support the use of temozolomide for primary spinal GBM.

**Figure 2:**
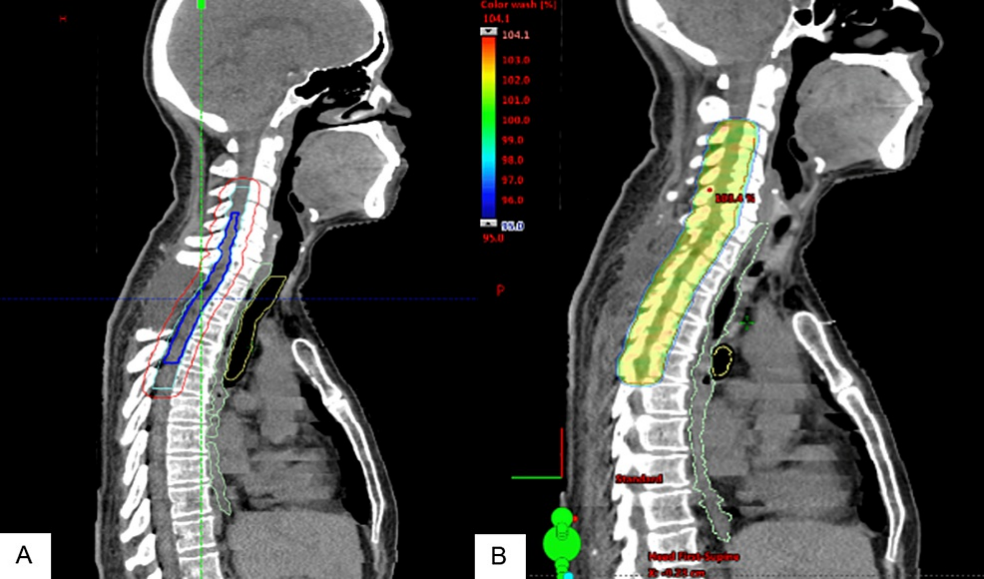
Radiation treatment plan (A) Sagittal view showing planning target volume in red, clinical target volume in cyan, and gross tumor volume in blue.  (B) VMAT plan showing dose color wash.

The patient was informed of the disease's incurable nature and poor prognosis, and high-dose palliative radiation therapy was recommended to control disease progression. Thirty-eight days postoperatively, the patient received a course of radiation using the volumetric modulated arc therapy (VMAT) technique (Figure 2). A prescription dose of 50 Gy in 25 fractions was chosen to maximize the dose to the tolerance of the spinal cord. The gross tumor volume was contoured using the pre-operative and post-operative MRI with a 2 cm craniocaudal margin for the clinical target volume limited within the spinal canal. An isotropic 7 mm planning target volume (PTV) margin was used per institutional protocol. The planning aim was to cover at least 95% of the PTV with 98% of the prescribed dose. The Quantitative Analyses of Normal Tissue Effects in the Clinic (QUANTEC) dose constraints were followed for the organs at risk. During the treatment course, she experienced mild fatigue with no other acute side effects or changes to her neurological status.

Thirteen days after completing radiation therapy, the patient was admitted for progressive dyspnea and management of hypoxemia. A chest X-ray showed reduced lung volumes with diffuse patchy airspace disease throughout both lungs and small bilateral pleural effusions. Differential diagnoses included diaphragmatic paralysis secondary to tumor progression, pneumonia, and radiation pneumonitis. Due to the patient’s rapidly declining health and known poor prognosis, the patient was transferred to palliative care and expired eight days after hospital admission, three months after pathologic diagnosis.

Case two

A 44-year-old male firefighter initially presented with progressive lower back pain that appeared near the end of the day. He also noticed weakness in his right leg during stair-climbing and sit-to-stand transitions. He was otherwise healthy, a nonsmoker, and had no comorbidities.

**Figure 3:**
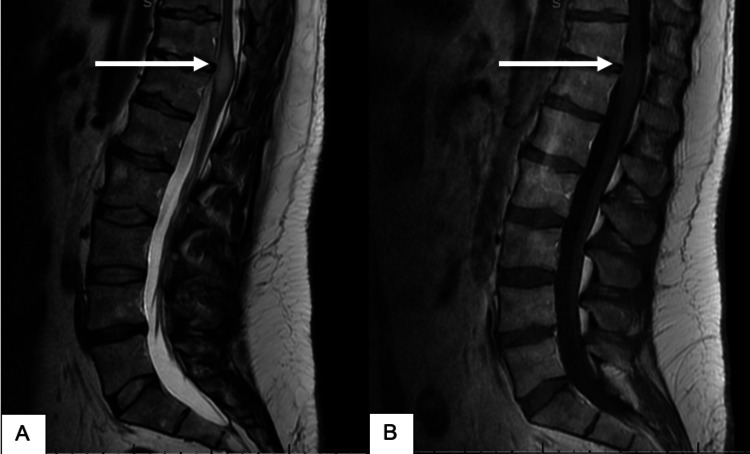
MRI of the lumbar spine pre-operatively, sagittal view (A) T2-weighted and (B) T1-weighted post-contrast images showing a non-enhancing intramedullary mass (white arrows) at the T11-12 level.

**Figure 4:**
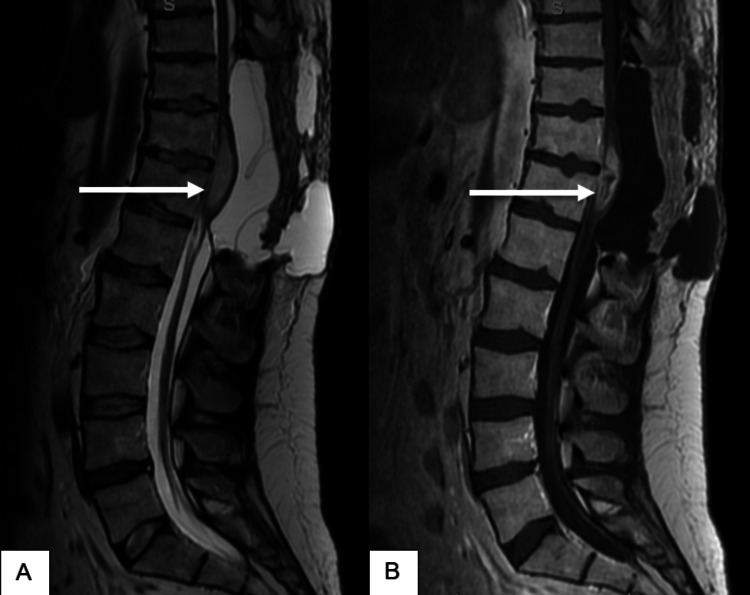
MRI of the lumbar spine post-subtotal removal of tumor, sagittal view (A) T2-weighted and (B) T1-weighted post-contrast images showing residual enhancing intramedullary mass (white arrows) at the T11-12 level.

MRI of the lumbar spine demonstrated a non-enhancing intramedullary mass at the T11-12 level, which was T2-hyperintense and T1-isointense (Figure 3). Seven days after the onset of symptoms, he underwent microsurgical subtotal removal of the spinal cord tumor. Pathology review of the resected specimen established the diagnosis of primary spinal GBM, IDH-wild type, histone 3 mutation not reported. Computed tomography (CT) scan of the chest, abdomen, and pelvis was negative for distant metastases, while a follow-up MRI of the brain was also negative for intracranial disease. MRI of the entire spine done one-month post-operatively demonstrated residual enhancing intradural lesion at T11-T12 (Figure 4). The neurologic evaluation demonstrated a significant weakness in the right lower extremity. Sensory evaluation showed more impaired sensation on the right side compared to the left side.

Two months postoperatively, he developed progressive symptoms, and a follow-up MRI demonstrated residual enhancing mildly heterogeneous solid mass at the T11-12 level measuring 4 cm in length and 1.2 cm in greatest diameter. After a thorough discussion, the patient decided to undergo gross total resection of the intramedullary spinal cord tumor to try to extend his life regardless of the deficits he would incur with radical resection. Sixty-three days after the onset of symptoms, the patient underwent gross total resection of the residual spinal cord tumor. Following surgery, the patient had no strength in his lower extremities and no sensation below the level of the umbilicus. He used a Foley catheter and had fecal incontinence. The pathology from the initial biopsy was reviewed at the Mayo Clinic and confirmed to be primary spinal GBM.

**Figure 5:**
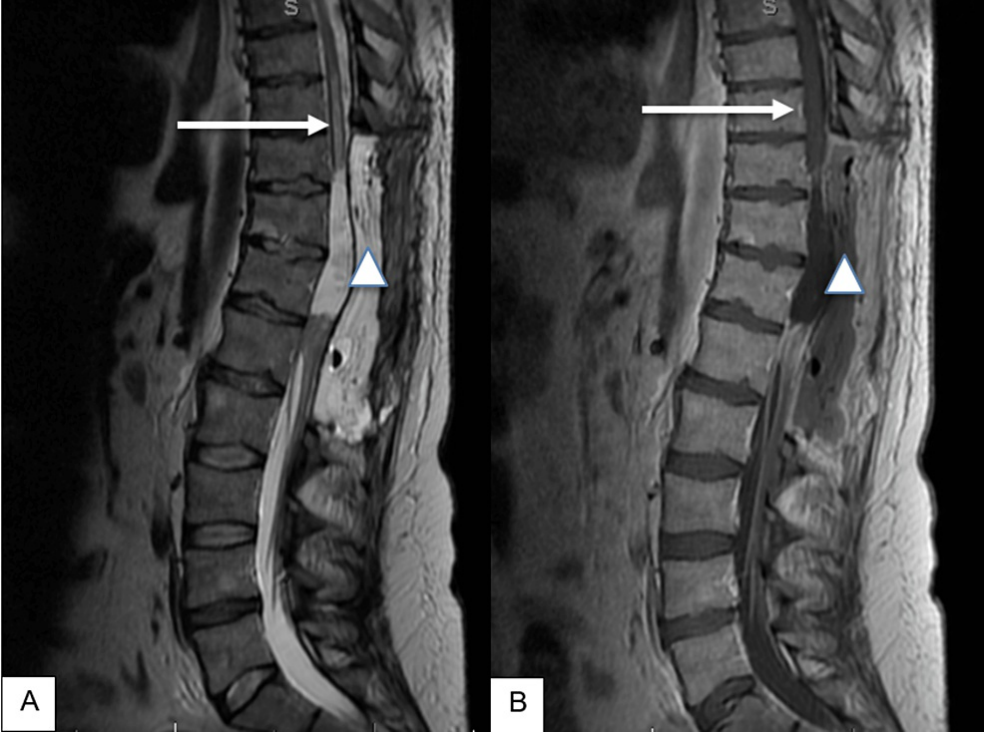
Post-operative MRI of the lumbar spine, sagittal view (A) T2-weighted and (B) T1-weighted post-contrast images showing a syrinx from the inferior border of T8 to mid-T10 (arrows) with a seroma (triangles) from the inferior border of T9 to L2 level.

**Figure 6:**
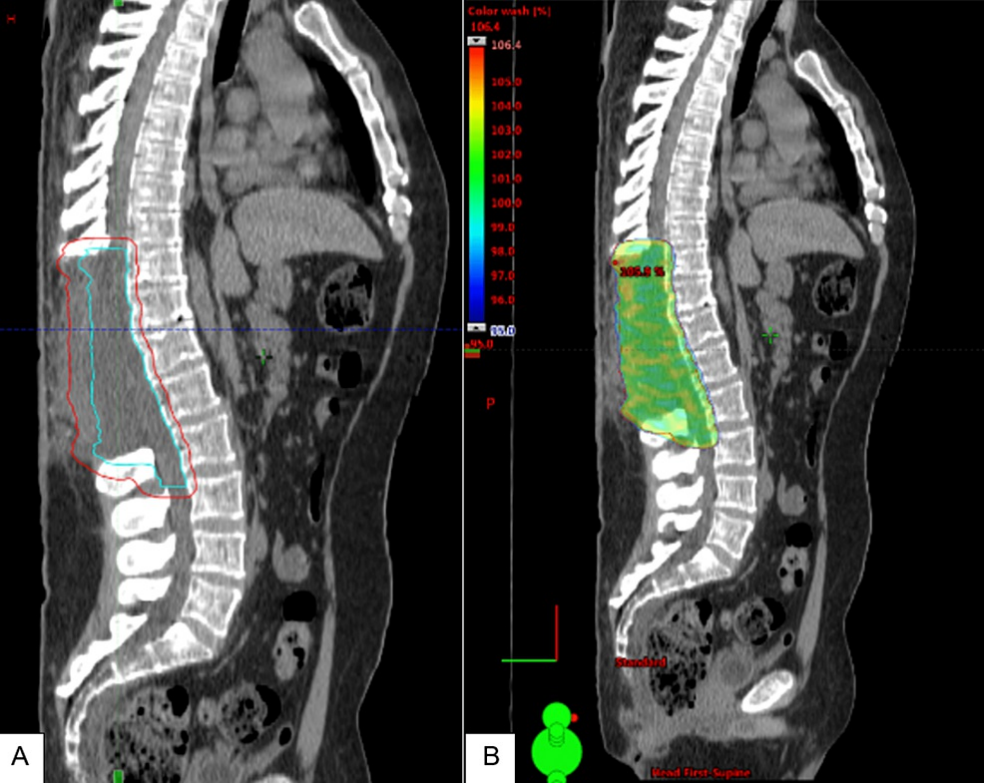
Radiation treatment plan (A) Sagittal view showing primary target volume in red and clinical target volume in cyan. (B) VMAT plan showing dose color wash.

His case was discussed at a multidisciplinary neuro-oncology conference where an imaging review noted some suspicion of possible residual disease in the lower end of the resection area, with a higher potential for residual disease inferior to the gross tumor (Figure 5). After discussion, the group decided to proceed with adjuvant radiation therapy. Three and a half months after initial resection, the patient received adjuvant radiation therapy to a total dose of 59.4 Gy in 33 fractions using the VMAT technique, including the volume 5 cm above and below the original lesion (Figure 6). The absence of function to preserve allowed the use of full-dose radiation therapy. The planning aim was to cover at least 95% of the PTV with 98% of the prescribed dose. The dose constraint for both kidneys was a mean dose < 15 Gy, the small bowel was V45 < 195 cc and the liver was a mean dose < 30 Gy. The role of systemic chemotherapy, specifically with temozolomide, was uncertain because of the rare presentation of GBM in the spinal cord and the lack of documented evidence of efficacy. However, considering the patient’s young age, tumor location, absence of comorbidities, and extreme motivation to seek treatment, chemotherapy was recommended with temozolomide 75 mg/m^2^ daily concurrent with radiation treatment. 

The patient experienced mild to moderate nausea and vomiting during the first few weeks of his radiation therapy, which were managed with anti-emetics. He was hospitalized throughout his treatment to undergo physical rehabilitation. There was no significant dermatitis, pain, or diarrhea.

**Figure 7:**
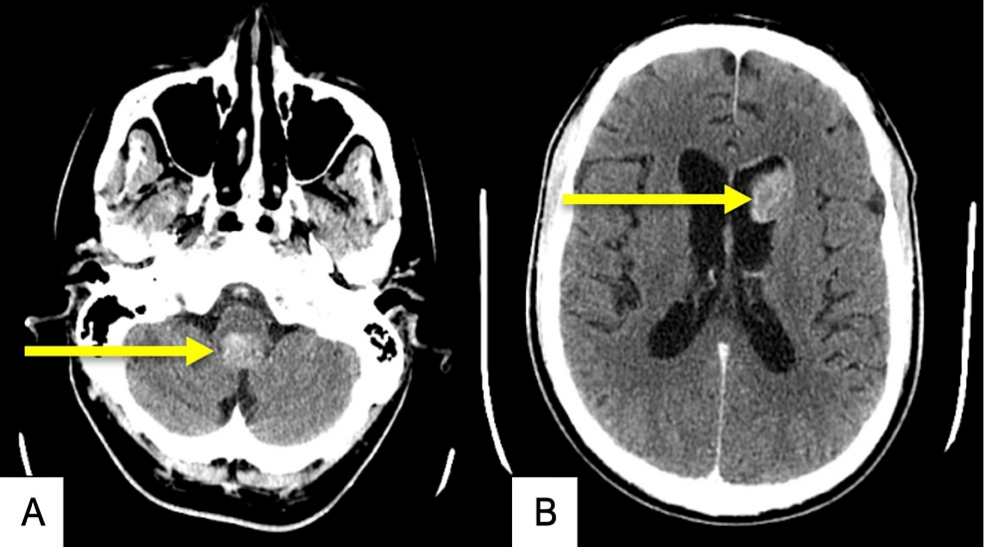
CT scan of the brain, axial view (A-B) Contrast scans showing two enhancing soft tissue masses (yellow arrows) in the base of the fourth ventricle and anterior horn of the left lateral ventricle

Four weeks post-radiation therapy, he started the first of a planned six cycles of adjuvant temozolomide, 150 mg/m^2^, for five days every 28 days. At six weeks post-radiation therapy, he still experienced persistent nausea. Follow-up MRI scans were pending; however, the patient had a seizure in the interim. A cranial CT scan revealed two enhancing soft tissue masses within the anterior horn of the left lateral ventricle (1.6 cm x 2.4 cm x 2.5 cm) and at the base of the fourth ventricle (2.6 cm x 2 cm x 1.7 cm) with hydrocephalus and diffuse dural enhancement suggestive of intracranial disease progression (Figure 7). A small focus of high density was noted within the tumor involving the fourth ventricle suggestive of a small bleed. Further investigations were not performed, as the patient’s condition deteriorated quickly, and he expired three days after the seizure, 7.4 months after the pathologic diagnosis.

## Discussion

Primary spinal GBM often affects adolescents and young adults with a mean age of 25 years and slight female predominance [3,4]. The patients in this series were both greater than 40 years old, which is older than the median age of 22 years, from a recent systemic review by Beyer et al. [5]. Their presentation was concordant with injury to the involved spinal level manifesting as motor-sensory deficits and back pain, similar to previous reports [6]. The first patient’s tumor was located in the cervicothoracic spine, which is the most common location for primary spinal GBM, while the second patient’s tumor was in the conus medullaris, which is a rare location [2,7,8]. The second case also progressed intracranially, despite aggressive resection of the primary spinal cord tumor. It is unknown what percentage of primary spinal GBM have synchronous or metastatic intracranial GBM, with few cases of intracranial metastasis previously published [9,10]. This highlights the importance of performing complete craniospinal imaging for patients suspected to have spinal glioblastoma to screen the entire neuraxis for disease.

Similar to intracranial glioblastomas, primary spinal GBM has a poor prognosis with a mean survival of approximately 10-12 months [2,11]. Higher survival rates were reported in a National Cancer Database study (2004 to 2016) with a median OS of 23.9 months [12] and a case report by Nagarajan et al. reporting a disease-free survival of 96 months [13]. While older age is an established poor prognostic factor for primary brain GBM, the prognostic role of age in primary spinal GBM is less clear due to mixed results in the literature. Konar et al. found that patients aged 18-65 years had better survival compared to patients aged <18 or >65 years old [6]. Adams et al. found that adult patients had markedly longer survival compared to pediatric patients [11]. On the other hand, Santi et al. reported that patients older than 40 years had shorter survival compared to younger patients [14]. The location of the tumor in the spine may also be a prognostic factor. Thoracic spine tumors were reported to have a better prognosis than cervical spine tumors, likely due to the possibility of more aggressive treatment in non-cervical spine regions [2].

The literature has conflicting reports on the benefits of surgery, radiation therapy, and chemotherapy for primary spinal GBM [2,6]; thus, no best treatment can be recommended based on current evidence. While the lack of evidence has led to the extrapolation of management from intracranial GBM, these generalizations have not led to improvements in outcomes over the last decade [11]. The second patient in this series did not experience a survival benefit despite having received aggressive trimodality treatment with gross total resection and postoperative radiation therapy with temozolomide, similar to previous studies [9,10,15]. 

As to the question of whether radical surgery benefits these patients, Timmons et al. reported in a systematic review that there is no significant difference in survival between patients treated with total resection versus subtotal resection [2]. Yanamadala et al. used subtotal resection in their single-institution study, and their reported one-year survival rate was 100%, with stable ASIA and Karnofsky Performance Scale scores at 3 months in 83% and 50% of patients, respectively [3]. 

A systematic review revealed that 65% of patients with primary spinal GBM received chemotherapy, with temozolomide being the most common drug at 70%. Other chemotherapies used in studies were nimustine, lomustine, ranimustine, cyclophosphamide, ThioTEP, dacarbazine, and PCV (procarbazine, lomustine, and vincristine). However, no significant difference was seen between patients treated with temozolomide versus other types of chemotherapy [2]. MGMT methylation status only has prognostic significance for supratentorial GBM [16]; thus, evidence on the benefit of temozolomide at present is limited to intracranial GBM. 

About 85% of patients with primary spinal GBM receive radiation therapy [2]. The radiation doses used in this series were 50 Gy and 59.4 Gy, consistent with doses reported in the literature ranging from 43.2 Gy to 65 Gy [2,13]. However, while there are studies describing the benefits of radiation therapy [6,11,17], other studies suggest that neither radiation therapy nor radiation dose is associated with survival [7,12]. Similarly, the use of more conformal techniques, such as VMAT, in this series did not confer better outcomes. The radiation therapy treatment volumes to be included were also not thoroughly reported in the published literature, with the majority employing limited volumes around the gross tumor or post-operative bed. Moreover, there is a paucity of studies on the quality of life in patients with primary spinal GBM, mostly limited to pediatric patients post-operatively. In cancer diagnoses with no established standard of care, quality-of-life outcomes may have more clinical significance in helping patients and clinicians make treatment decisions.

The time to onset of treatment may also be critical in understanding the prognosis and management of primary spinal GBM. A systematic review reported the median time to the start of surgery, radiation therapy, and chemotherapy to be between 1 to 2 months after diagnosis [2]. For this case series, radiation therapy was done 38 days and 110 days from diagnosis, with initial surgeries performed within one week of symptoms. Confounding comorbidities and complications due to immobility or poor health can likewise compete with the primary cancer diagnosis as the cause of mortality.

The lack of significant survival improvements with current treatments has paved the way for investigations on the role of immunotherapy. A case report of GBM metastatic to the spine showed the efficacy of intracavitary infusions of IL13Rα2 targeted CAR T cells, causing 100% regression of all gross disease with a disease-free period of 7.5 months before a recurrence in new sites [18]. Another case report using anlotinib for a primary spinal GBM with FGFR3 mutation resulted in a disease-free interval of more than 10 months [19]. The genomic landscape of high-grade spinal astrocytomas is now understood to be different from intracranial neoplasms and may include mutations in H3K27M, TP53, and TERT promoters. However, immunotherapy research in primary spinal GBM remains limited due to the small number of specific antigens identified, neurotoxicity of drugs to the spinal cord, scarcity of cases, and difficulty obtaining sufficient tissue for immunologic studies [20].

## Conclusions

The clinical presentation of patients in this series was consistent with the involved spinal level where the tumors were located. Their age at presentation is older than the majority of patients published in the available literature, which may have influenced their poor prognosis. Craniospinal MRI is crucial in determining the presence of disease and should be considered in all patients due to the possibility of concomitant intracranial disease. However, the absolute utility of surgery, chemotherapy and radiation therapy remains unclear. Even with aggressive treatment, patient survival remained poor from the onset of symptoms, mirroring the unfavorable prognosis of intracranial GBM due to the natural history of the disease. Decision-making for the management of primary spinal GBM should be tailored to the patient- and tumor-related factors as part of a patient-centered multidisciplinary team.
